# Disseminated cryptococcosis with meningitis, peritonitis, and cryptococcemia in a HIV-negative patient with cirrhosis: a case report

**DOI:** 10.1186/1757-1626-2-170

**Published:** 2009-10-28

**Authors:** Baligh Ramzi Yehia, Michael Eberlein, Stephen D Sisson, David N Hager

**Affiliations:** 1 Department of Medicine, Johns Hopkins University School of Medicine, 1830 East Monument Street, Suite 9020, Baltimore, Maryland, USA

## Abstract

**Introduction:**

*Cryptococcus neoformans *is an encapsulated yeast that causes serious infections in immunocompromised populations. The majority of cases occur in HIV-infected individuals. Disseminated disease is uncommon, and very rarely includes peritonitis.

**Case presentation:**

We report a case of a 41-year-old, HIV-negative, Caucasian man with alcoholic liver cirrhosis who presented with fever and seizure. Disseminated cryptococcosis with meningitis, peritonitis, and cryptococcemia was diagnosed, and despite adequate treatment the patient developed multi-system organ failure and eventually expired.

**Conclusion:**

Disseminated cryptococcosis, particularly with peritonitis, is an uncommon manifestation of *Cryptococcus neoformans *infection. Liver cirrhosis serves as a risk factor for disseminated disease in HIV-negative patients. A high clinical suspicion and early initiation of therapy is needed to recognize and treat patients effectively.

## Introduction

*Cryptococcus neoformans *is a ubiquitous encapsulated yeast that predominately causes significant infections in immunocompromised individuals, with eighty to ninety percent of all cases occurring in those with HIV infection [1,2]. The most common sites of infection are the central nervous system (CNS) and lungs [1-4]. Disseminated disease is uncommon and when present almost always occurs in HIV-infected patients [2]. Cryptococcal peritonitis occurs even less frequently then disseminated disease and is considered a rare manifestation of cryptococcosis [1]. Here, we present a case of disseminated cryptococcosis with meningitis, peritonitis, and cryptococcemia occurring in a HIV-negative patient with alcoholic liver cirrhosis.

## Case presentation

A 41-year-old Caucasian man presented to the emergency department with a 1-month history of jaundice, hallucinations, and ataxia. He was initially treated for acute alcoholic hepatitis with pentoxifylline and steroids, hepatic encephalopathy with lactulose, and started on empiric therapy for spontaneous bacterial peritonitis (SBP) with ceftriaxone. His mental status returned to baseline, but he continued to have worsening liver and renal failure. Hepatorenal syndrome was diagnosed and he was started on octreotide, midrodine and albumin.

His medical history was notable for an ischemic stroke with mild residual deficit, gastric bypass surgery, uncomplicated lumbar spinal fusion surgery, and alcohol induced cirrhosis of the liver. Social history suggested that he consumed approximately one pint of vodka per day. Family history was noncontributory. He was not taking any medications at home.

On hospital day 22, he sustained a tonic-clonic seizure, which lasted 4 minutes. Given his prolonged post-ictal state and low blood pressure he was transferred to the intensive care unit (ICU). Evaluation at that time revealed a severely obtunded and jaundiced man. Vital signs showed a temperature of 38.4°C, heart rate of 92 beats/min, blood pressure of 109/62, and respiratory rate of 19 breaths/min. Oxygen saturation by pulse oximetry was 95% on room air. Head and neck examination revealed scleral icterus and a supple neck. Diffuse coarse crackles were noted on auscultation of the chest. The cardiac rhythm was regular with a normal rate. No murmurs were appreciated. His abdomen was markedly distended, consistent with ascites. Guarding, rebound tenderness, and organomegaly were not appreciated. Neurologic examination revealed sluggish pupils, a weak gag reflex, and movement of the extremities only in response to painful stimuli. No asymmetry was appreciated.

Laboratory studies were obtained and revealed a white blood cell (WBC) count of 62 × 10^3 ^cells/mm^3^, with 89% polymorphonucleated cells (PMNs), and a platelet count of 112 × 10^3 ^per mm^3^. His INR was 1.5. An extended metabolic and liver panel demonstrated: sodium 127 mEq/L, BUN 63 mg/dL, creatinine 6.8 mg/dL, total bilirubin 39.8 mg/dL, AST 201 U/L, ALT 109 U/L, and alkaline phosphatase 349 U/L. Ammonia was 38 μmol/L. Arterial blood gas showed a pH of 7.33, PCO_2 _of 35 mm Hg, PO_2 _of 184 mm Hg, and HCO3 concentration of 15 mEq/L on 40% oxygen. HIV antibody was found to be negative and HIV-1 RNA undetectable.

Given his seizure, fever, and leukemoid reaction, he was started on empiric therapy with cefepime, vancomycin, ampicillin, and acyclovir. Levetiracetam (Keppra) was initiated for seizure control. An MRI of the brain was negative for any evidence of intracranial bleeding or other space occupying lesions. An EEG was unremarkable. Lumbar puncture was attempted, but unsuccessful due to the patient's prior spine surgery. Paracentesis revealed ascitic fluid with a WBC of 797 per mm^3 ^with 81% PMNs, despite therapy with ceftriaxone. Ascites gram stain, bacterial and fungal cultures were negative. Because of his progressive clinical deterioration amphotericin was added to his regimen on hospital day 23.

**Figure 1:**
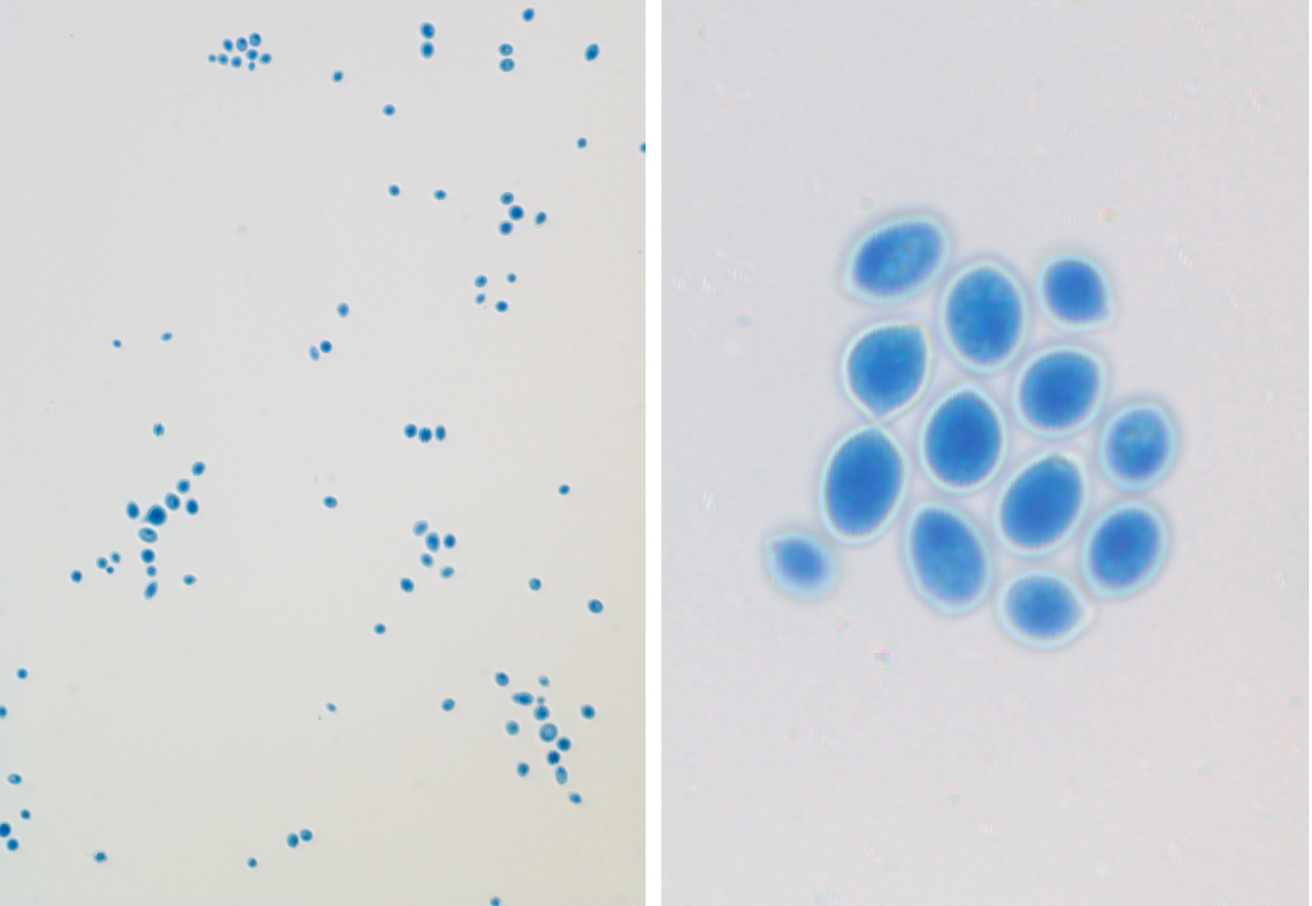
**Lactophenol Cotton Blue stain of CSF culture noted encapsulated yeast consistent with *Cryptococcus neoformans *(× 100, × 400)**.

Lumbar puncture under fluoroscopy was performed on hospital day 24 and cerebrospinal fluid (CSF) analysis revealed 104 WBCs per mm^3 ^with 98% PMNS, glucose 47 mg/dL, and protein 84 mg/dL. CSF gram stain demonstrated yeast and culture grew *Cryptococcus neoforman *(Figure 1). CSF cryptococcal antigen was also positive. Though initially negative, blood cultures also grew *C. neoformans*. Flucytosine was added to his medical regimen for better CSF coverage.

The patient was diagnosed with disseminated cryptococcosis with meningitis, peritonitis, and cryptococcemia. Meningitis and cryptococcemia, as demonstrated by positive *Cryptococcus neoformans *cultures, and peritonitis, based on peritoneal fluid leukocytosis in the setting of low suspicion for SBP. The leukemoid reaction was attributed to alcoholic hepatitis, as cryptococcemia is not a known cause and no other infections were identified. Unfortunately, the patient continued to deteriorate with refractory hypotension and multi-system organ failure. Given his overall clinical picture and poor prognosis, the patient's family ultimately elected to withdraw life supporting measures. The patient expired soon thereafter.

## Discussion

*Cryptococcus neoformans *is an encapsulated yeast that predominately affects immunocomprimised individuals. In addition to HIV infection, immunosuppressive medications, solid-organ transplantation, chronic organ failure (renal and liver), hematologic malignancy, chronic lung disease, and rheumatologic disorders can also predispose individuals to this infection [3].

Meningoenecephalitis and pulmonary infiltrates are the two most common manifestations of cryptococcal disease [1,2,5]. Disseminated cryptococcosis is defined by 1) a positive culture from at least two different sites, or 2) a positive blood culture [5]. In a series of 33 HIV-negative patients with disseminated disease, cirrhosis was the most frequent predisposing condition and was associated with a grave prognosis [5,6]. Leukocyte impairment, complement dysfunction, and decreased opsonin activity are believed to predispose cirrhotic patients to cryptococcosis [1].

Cryptococcal peritonitis is an unusual manifestation of *C. neoformans *[1]. Infection of ascitic fluid accounts for less than 5% of all cryptococcosis cases in HIV-negative patients [1,4-6]. When liver cirrhosis is jointly considered, the prevalence of cryptococcal peritonitis is exceedingly rare [1,4-6]. Our literature review only identified 19 cases of cryptococcal peritonitis in HIV-negative patients with liver cirrhosis [1,4-6].

The clinical presentation of disseminated cryptococcosis is variable and depends on the organ systems involved. *C. neoformans *has been identified in cultures from blood, CSF, sputum, ascites, urine, bone marrow, and skin [2,5]. A diffuse maculo-papular rash may be an important diagnostic clue indicating disseminated disease [7]. Central nervous system (CNS) involvement is the most common manifestation of disseminated cryptococcosis [5]. Patients often present in a subacute manner with headache and fever. Other symptoms can include seizures, confusion, dementia, and bizarre behavior. Cerebral edema leading to elevated intracranial pressure can cause blurred vision, diplopia, confusion, hearing loss, and severe headaches [8]. In patients with cryptococcal peritonitis, diagnosis can often be delayed due to lack of specific signs and symptoms and low clinical suspicion among healthcare providers [1]. Abdominal pain, increased abdominal girth, fever, and dyspnea are typical complaints of patients with cryptococcal peritonitis [1,4].

In patients with CNS disease, imaging of the brain is usually unremarkable and lumbar puncture is often necessary to establish the diagnosis. CSF analysis typically reveals a lymphocytic pleocytosis with elevated protein and decreased glucose. Opening pressure during lumbar puncture is often elevated to values greater than 20 cm H_2_O [8]. India ink staining will identify C. *neoformans *in the CSF of patients with HIV greater than 90% of the time and in more than 50% of infected patients without HIV [9]. The CSF cryptococcal antigen is almost universally positive in established CNS infection, but may be negative very early in a patient's clinical course [8,9]. Similar to CNS infection, diagnosis of peritoneal disease relies on analysis of ascites fluid and not imaging studies. Diagnosis can be difficult, requiring multiple ascites fluid samples and prolonged culture time [10]. Ascitic fluid usually demonstrates a low protein level and moderately elevated WBC count [1]. Cryptococcal antigen testing can be performed on ascites fluid, although limited studies exist on its use and utility [1]. The diagnostic gold standard for *C. neoformans *infection is growth of the organism in culture from an otherwise sterile site [8,9].

Treatment regimens are determined by anatomic site of infection and host immune status. Though no specific guidelines exist for the treatment of cryptococcal peritonitis, recommendations for the treatment of CNS disease are well established [11]. Immunocompetent patients with cryptococcal meningitis should be treated with amphotericin B and flucytosine for 6-10 weeks, or amphotericin B and flucytosine for 2 weeks followed by fluconazole (400 mg/d) for a minimum of 10 weeks [11]. For immunosuppressed patients without HIV infection, it is recommended that cryptococcal meningitis be treated with amphotericin B +/- flucytosine for 2 weeks followed by 8-10 weeks of fluconazole (400-800 mg/d), and then fluconazole (200 mg/d) for an additional 6-12 months [11].

Patients with isolated cryptococcal meningitis typically improve with appropriate therapy. Depressed levels of consciousness, high CSF cryptococcal antigen titer, and cryptococcemia are associated with a poor prognosis [9]. An opening pressure greater than 25 cm H_2_O is the most important prognostic factor and is associated with increased morbidity, including diminished mental capacity, vision loss, cranial nerve palsies, and hydrocephalus [8]. Prognosis for HIV-negative patients with disseminated cryptococcosis and liver cirrhosis is poor, with 30 day mortality documented at 100% in one study [5]. In patients with cryptococcemia, liver cirrhosis was the strongest independent predictor of 30-day morality (hazard ratio 16.3, 95% CI 2.6-101.7, p = 0.003), even when compared to HIV infection [6].

## Conclusion

In summary, disseminated cryptococcosis, particularly with peritonitis, is an uncommon manifestation of *C. neoformans *infection. Liver cirrhosis serves as a risk factor for disseminated disease in HIV-negative patients and is associated with a grave prognosis. A high clinical suspicion and early initiation of therapy is needed to recognize and treat patients effectively.

## Consent

Unfortunately, the patient expired and despite multiple attempts to contact the family, the next of kin was not available. The patient case is presented anonymously, and there is no reason to think that the patient or their family would object to publication.

## Competing interests

The authors declare that they have no competing interests.

## Authors' contributions

BY, ME, and DH were involved in the direct care of the patient, and contributed to the literature search, data collection, data analysis, and manuscript preparation. SS was involved in the literature search, data analysis, and manuscript preparation. All authors have read and approve of the submitted manuscript.
